# Factors Associated With Third Molar Eruption in the Assamese Inhabitants of Northeast India

**DOI:** 10.7759/cureus.93165

**Published:** 2025-09-25

**Authors:** Putul Mahanta, Rejum Ronya, Subu Sumpi, Neelutpal Bora, Madhab C Rajbangshi, Krishangi Kashyap, Kumar P Pratim

**Affiliations:** 1 Forensic Medicine and Toxicology, Nalbari Medical College and Hospital, Nalbari, IND; 2 Surgery, Tomo Riba Institute of Health and Medical Sciences, Naharlagun, IND; 3 Orthodontics, Regional Dental College, Guwahati, IND; 4 General Surgery, Nalbari Medical College and Hospital, Nalbari, IND; 5 Pediatric Nursing, Nalbari Medical College and Hospital, Nalbari, IND; 6 Forensic Medicine, Gauhati Medical College, Guwahati, IND

**Keywords:** body mass index, chronological age, dental age, sociodemographic factor, third molar eruption, third molar teeth

## Abstract

Background

Genetic, nutritional, and regional factors should be considered when designing a technique to estimate age based on third molar eruption (TME), as they influence the eruption process of teeth. This study aims to investigate the sociodemographic characteristics associated with TME, as well as the average age of eruption of third molar teeth (M3) in Assamese individuals.

Methodology

This study included 753 participants, aged 14 to 26 years, comprising both rural and urban residents from the north-eastern Indian state of Assam. TME stages were determined using a conventional clinical dental examination. The participants underwent detailed medical screenings to determine their individual height and weight, calculate their body mass index (BMI), and rule out any obvious congenital or developmental abnormalities based on the clinical physical examination.

Results

Of the 753 participants, 302 (40.1%) had either partial or complete eruption of the third molars. The mean age of non-eruption was lower in males (mean ± standard deviation (SD) = 16.80 ± 2.11) than that of females (mean ± SD = 17.91 ± 2.25). Among males, the mean BMI differed considerably between the third molar erupted and non-erupted groups (p = 0.025). Additionally, the non-eruption of third molars was observed to be substantially higher among urban males (p < 0.01) and underweight females (82.6%, n = 38/46). Females had an early TME in both upper jaws, with a mean age of 19.88 (±3.36) years, compared to 20.00 (±2.27) years for males. In contrast to females, who had an average age of 19.24 (±1.90) years, males experienced early tooth loss in both mandibular jaws at an average age of 18.30 (±2.23) years.

Conclusions

Dental age estimated by the TME status is substantially associated with chronological age. Considerable variability exists in the TME status between genders among the inhabitants of this region. Various sociodemographic variables influence an individual’s TME status. Further community-based, elaborated studies are required to generalize these findings.

## Introduction

Age diagnosis is a vital task often required by forensic medicine specialists, as well as other medical professionals. In criminal investigations, forensic age assessment is crucial for identifying unknown individuals and reducing the number of potential suspects [1]. It may be challenging for medical experts to determine an individual’s age for identification purposes due to the wide range of individual variation. Tooth development is a crucial biological indicator for approximating chronological age in anthropology and research [2]. Additionally, understanding the basic underpinnings of typical human tooth eruption processes aids in the precise identification of eruption abnormalities and guides possible preventive or intervention measures.

Although the time taken for permanent teeth to appear varies, this stage is a crucial developmental milestone that can be used to track a child’s progress toward adolescence. Due to their impact on the eruption process of teeth, genetic, dietary, and regional factors should be considered when developing a technique to estimate age based on third molar eruption (TME) [3]. Variations in the average ages of the third molar teeth (M3) root stages have been reported among different ethnic groups [4]. The stages of tooth eruption can also be accelerated by nutritional status. Studies suggest that TME is correlated with an individual’s body mass index (BMI). Children who are overweight tend to experience early eruption, whereas chronic malnutrition in children causes a delayed eruption [5,6].

In the present study, the tooth eruption stages were investigated by a thorough clinical dental examination. Numerous facets of the eruption process have been investigated in studies on human tooth eruption. In contrast to experimental research, human studies are frequently clinical and radiographic in nature rather than histological [7]. Radiographic imaging is essential for the thorough assessment of M3, providing information on its angulation, impaction, and proximity to its neighboring structures. Whereas, in resource-limited research settings with large sample sizes, clinical dental examination provides ease in patient-centered assessment, rapid functional and pathological evaluation, cost-effectiveness, and safety considerations. The present study limits its observations to clinical dental eruption, specifically the intraoral phase of tooth eruption, during which the tooth becomes clinically apparent in the mouth and moves toward occlusal contact [8,9].

In the entire dentition process, M3 development is the primary indicator used to estimate an individual’s age after puberty. Region-specific age determination based on M3 growth stages needs population-specific standards due to high interethnic variation [10]. Despite being a critical indicator for approximating biological age, research on TME and its associated factors is scarce in this region. The present study hypothesized that various factors influence the eruption status of M3 in the Assamese population. The primary objective of the study was to evaluate the average age at eruption of M3 between genders and explore the factors affecting the TME status in the study population.

## Materials and methods

The present cross-sectional analytical research was conducted in the Department of Forensic Medicine and Toxicology at Tezpur Medical College and Hospital, Assam, from July 2014 to July 2018. The study’s reference population consisted of individuals between the ages of 14 and 26 years who resided in the north-eastern Indian state of Assam. Standard dental and physical examinations were conducted on 753 participants in this study, all of whom had a known chronological age. The participants’ chronological age was derived from their birth documents, including their passports, driver’s licenses, and birth certificates, after appropriate counselling and verification. The participants underwent detailed medical screenings to determine their individual height and weight, calculate their BMI, and rule out any obvious congenital or developmental abnormalities. The Human Ethics Committee of Tezpur Medical College and Hospital granted permission to conduct this study under (reference number: 02/IEC/IMC/14 dated 28-10-2014). Participants provided their informed consent before data collection.

Inclusion criteria

The study included healthy people who had been in Assam since birth, had no history of surgery or trauma to either jaw’s posterior quadrants, and were free of syndromic (Apert syndrome, ectodermal dysplasia, Hunter syndrome, etc.) and systemic (vitamin D-resistant rickets, hyperparathyroidism, etc.) disorders. Individuals free of known and suspected congenital abnormalities, growth retardation, and dental problems that might impair normal tooth development and growth were included in the study. To check for any visible signs of congenital abnormalities and pathological disorders that hinder tissue maturation, a general physical examination was conducted.

Exclusion criteria

Participants were excluded if they had impacted M3, hypothyroidism, Down syndrome, a history of M3 extraction, a family history of known congenital disorders, or a known congenital absence of the M3. As the eruption status was clinically examined in the current study, participants with impacted M3 were excluded from this study.

Sample size and sample selection

A total of 753 participants were included in the study. The sample size of the present study was calculated using the following formula: Sample size (n) = z^2^SD^2^/d^2^, where z = 1.96 at a 95% confidence level, SD = standard deviation (SD) in the population, and d = margin of error. Assuming a population SD of 0.56 and a d of 0.04, the estimated sample size for the study was 756.

The participants were selected purposively from two schools and two medical colleges in two different districts of Assam, namely, Kamrup (Metro) and Sonitpur. The proportion of samples from schools and medical colleges was 1:1.

Evaluation of TME status

**Figure 1:**
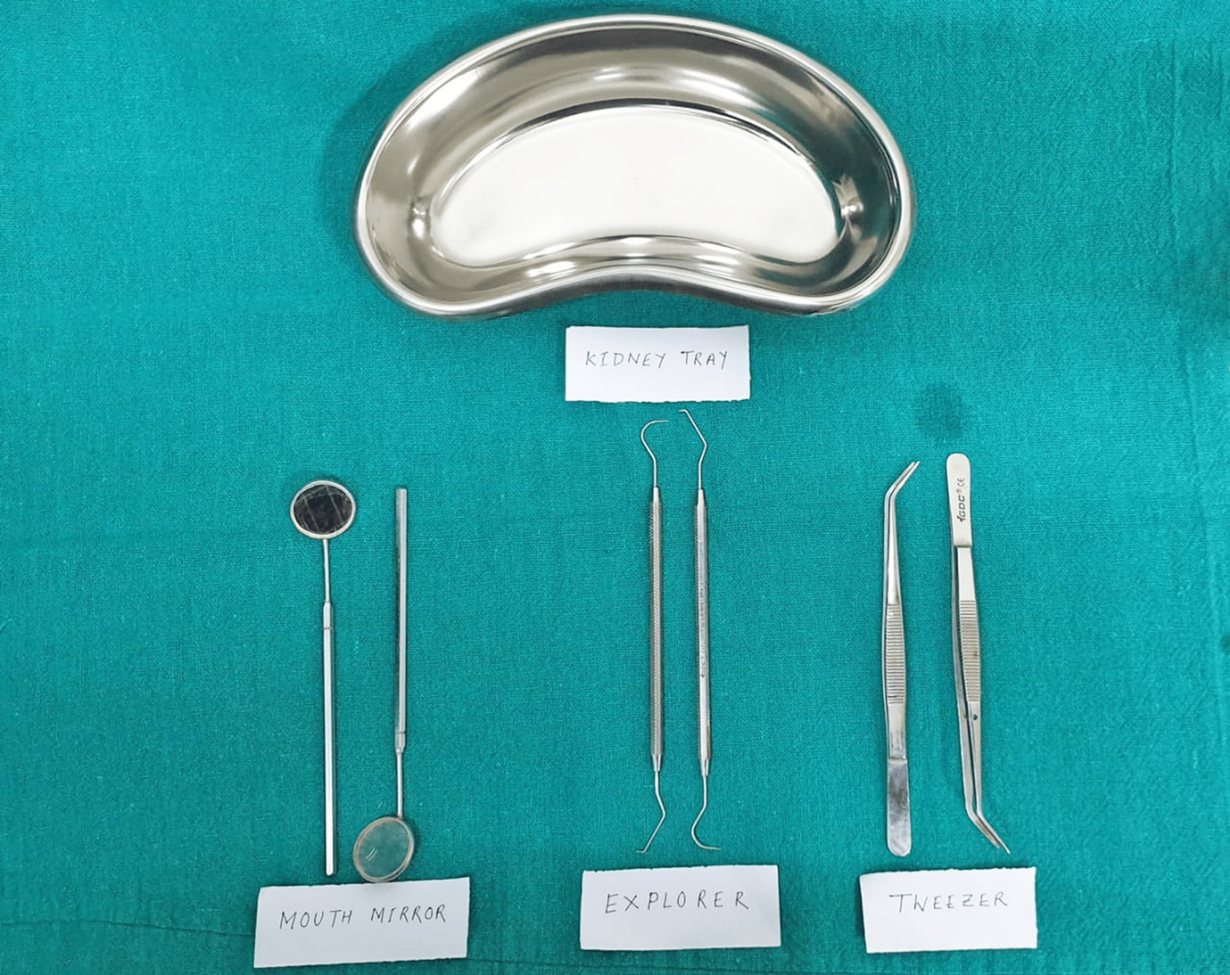
Diagnostic instruments used for a conventional dental check-up.

**Figure 2:**
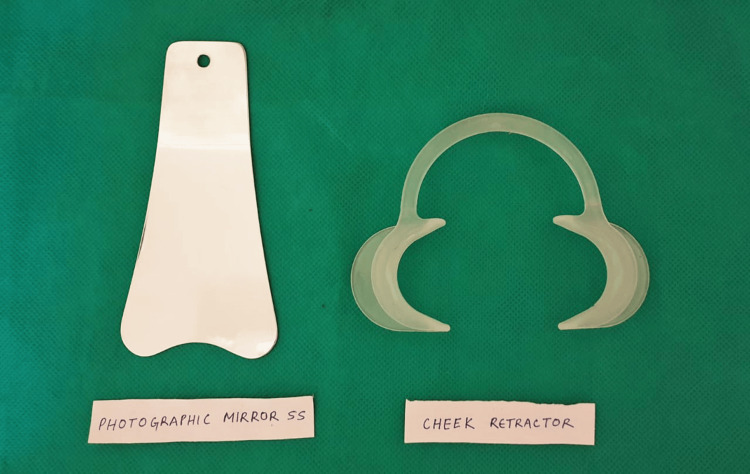
Diagnostic instruments used for a conventional dental check-up.

Using common dental instruments (Figures 1, 2), a clinical dental examination was conducted to assess the various stages of eruption of the M3.

A tooth was said to have “erupted” if evidence of the crown could be seen through the oral mucosa. TME status was categorized as “not erupted” if none of the M3s had erupted, “partially erupted” if any of the partially erupted M3s had visibly erupted but not all four, and “completely erupted” if all four M3s had erupted at the time of the study.

Three study authors evaluated the TME stages using a standard clinical dental examination. In a sample of 75 (10% of the total sample) selected individuals, we discovered the interobserver agreement to be 100% (Cohen’s kappa). The perfect interobserver agreement in the present study was attributed to the examiners’ extensive clinical experience and the clear criteria for detecting tooth eruption used in this study. As the radiographic examination of the tooth was not performed in this study, it was not possible to evaluate the stages of dental calcification. To corroborate the timing of the TME among the local population, 14-year-old participants were included in this study.

Statistical analysis

The current study compared the participants’ TME status with sociodemographic factors to assess any potential relationships. Descriptive statistical methods, including frequency, percentage, mean ± SD, and standard error of the mean (SEM), were employed to analyze the distribution of the variables under investigation. Significant differences among different categorical and continuous variables were assessed using the chi-square test and Student’s t-test for two independent samples, respectively. The normality of the data was tested using the Kolmogorov-Smirnov test, which showed that the data were not normally distributed. However, as the t-test is reliable even for highly skewed distributions for large sample sizes, it was used to compare mean age and mean BMI among groups. The data analysis was performed by using Microsoft Excel (Microsoft Corp., Redmond, WA, USA) and the SPSS version 22 (IBM Corp., Armonk, NY, USA). A p-value <0.05 was considered significant.

## Results

**Figure 3:**
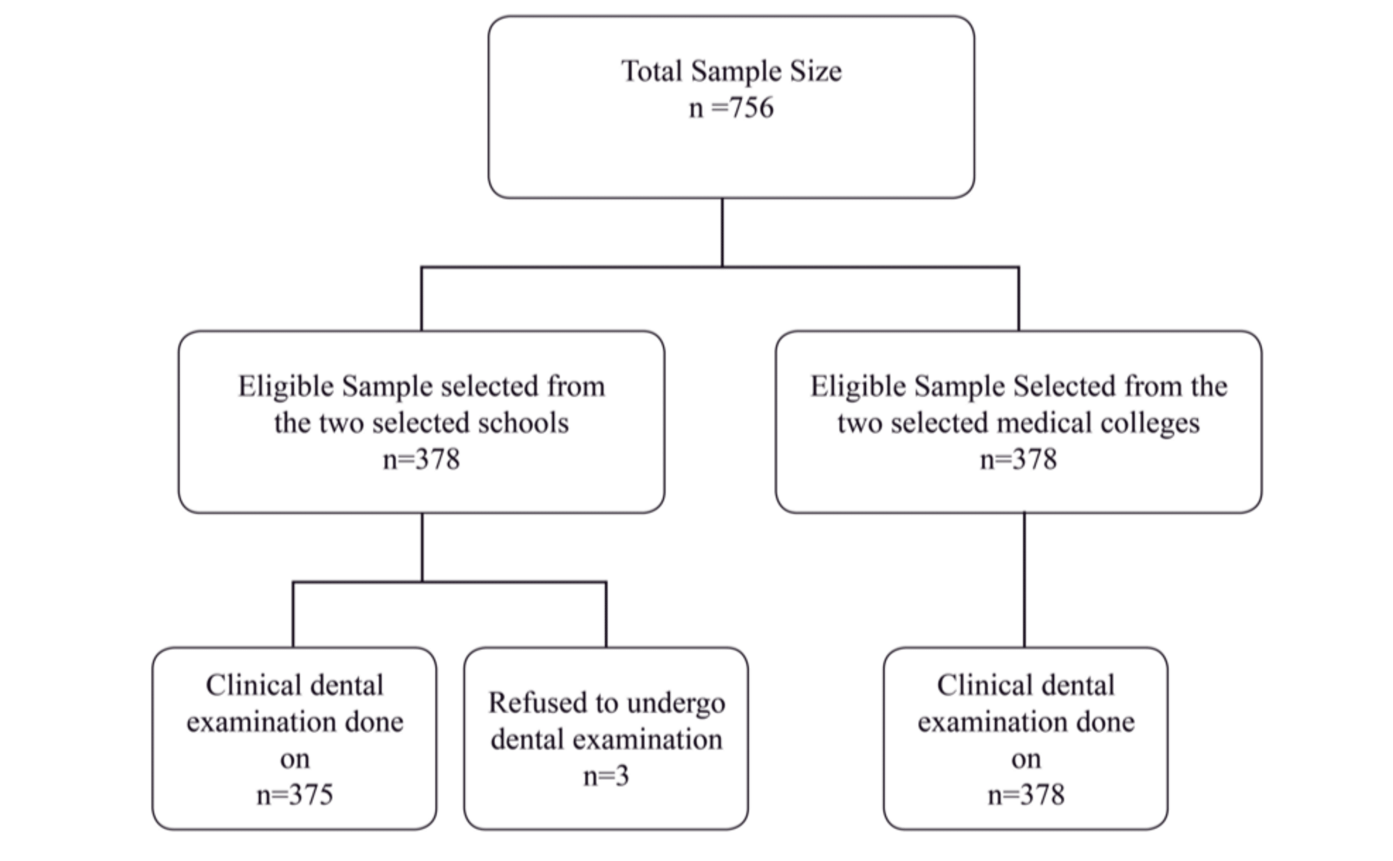
Study flow diagram.

Figure 3 shows a flow diagram illustrating the sample selection process employed in this study.

TME status

**Figure 4:**
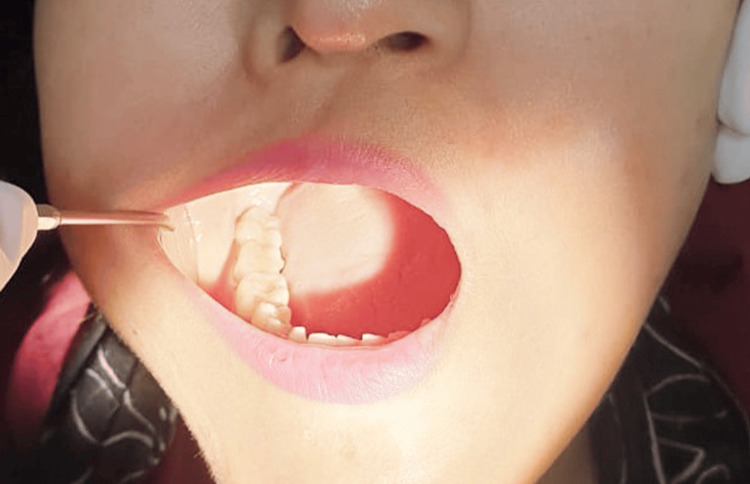
Intraoral view showing complete third molar eruption in the left quadrant.

**Figure 5:**
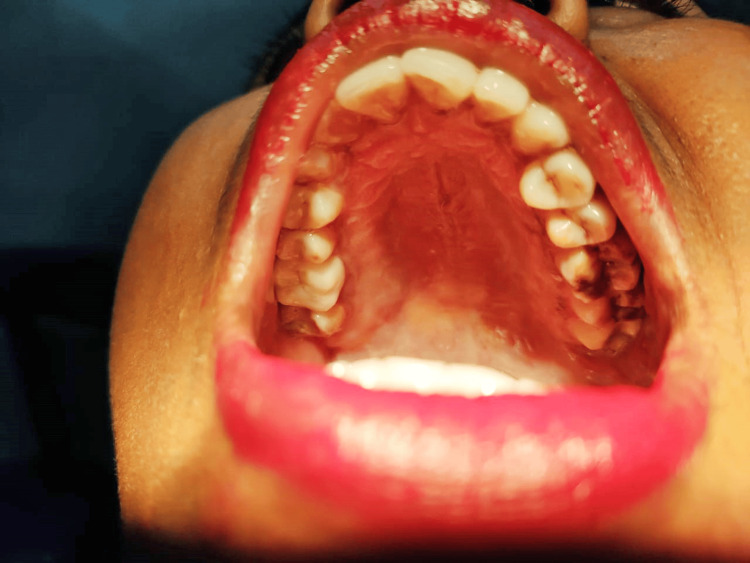
Intraoral view showing no eruption of the third molar.

A few cases showing complete eruption (Figure 4) and no eruption (Figure 5) status of M3 among the participants are presented for better understanding.

Socio-demographic pattern of participants

Out of the 753 participants, 302 (40.1%) had either partial or complete eruption of the M3, while 451 (59.9%) had no eruption. Among the participants, the majority, 456 (60.6%), were males. The incidence of TME with chronological age revealed that all 30 participants (100.0%) under the age of 14 years had no eruption. Eruption status differed significantly across different chronological age groups (chi-square value =128.2; p < 0.01). TME was significantly more prevalent in males (n = 197, 43.2%) and among rural participants (n = 102/181, 56.4%). Moreover, TME was least prevalent among the underweight participants (n = 82/111, 73.9%), indicating a highly significant association (p = 0.01) between BMI and TME (Table 1).

**Table 1:** Sociodemographic data of the participants as per third molar eruption. The data has been represented as frequency (n) and percentage (%); total sample (N) = 753. BMI: body mass index

Variables	Categories	Total	Third molar		Chi-square	P-value
			Not erupted (n = 451)	Erupted (n = 302)		
Chronological age	14	30	30 (100.0%)	0 (0.0%)	128.2	<0.01
	15	104	87 (83.7%)	17 (16.3%)		
	16	91	68 (74.7%)	23 (25.3%)		
	17	145	97 (66.9%)	48 (33.1%)		
	18	63	38 (60.3%)	25 (39.7%)		
	19	91	46 (50.5%)	45 (49.5%)		
	20	96	45 (46.9%)	51 (53.1%)		
	21	66	27 (40.9%)	39 (59.1%)		
	22	21	8 (38.1%)	13 (61.9%)		
	23	17	1 (5.9%)	16 (94.1%)		
	24	10	0 (0.0%)	10 (100.0%)		
	25	9	1 (11.1%)	8 (88.9%)		
	26	10	3 (30.0%)	7 (70.0%)		
Sex	Male	456	259 (56.8%)	197 (43.2%)	4.6	0.03
	Female	297	192 (64.6%)	105 (35.4%)		
Residential status	Rural	181	79 (43.6%)	102 (56.4%)	26.2	<0.01
	Urban	572	372 (65.0%)	200 (35.0%)		
BMI	Normal	452	263 (58.2%)	189 (41.8%)	11.1	0.01
	Underweight	111	82 (73.9%)	29 (26.1%)		
	Overweight	141	80 (56.7%)	61 (43.3%)		
	Obese	49	26 (53.1%)	23 (46.9%)		

Mean age and mean BMI for TME among participants

The mean age at eruption of TME among the 302 participants with TME was substantially higher than that of those with no eruption (p < 0.01). Males had a lower average age of non-eruption (16.80 ± 2.11 years) compared to females (17.91 ± 2.25 years). The overall mean BMI was also noted to be substantially different (t-test value = 3.04; p = 0.002) among the M3 erupted and non-erupted groups (Table 2).

**Table 2:** Gender-wise distribution of mean age and mean BMI for third molar eruption status. The data has been represented as frequency (n) and percentage (%); total sample (N) =753. SD: standard deviation; SEM: standard error of mean; BMI: body mass index

Variables	Total		Male		Female	
	Erupted (N = 302)	Not erupted (N = 451)	Erupted (N = 197)	Not erupted (N = 259)	Erupted (N = 105)	Not erupted (N = 192)
Chronological age						
Mean ± SD (SEM)	19.40 ± 2.65 (0.15)	17.27 ± 2.24 (0.10)	19.22 ± 2.69 (0.19)	16.80 ± 2.11 (0.13)	19.74 ± 2.54 (0.25)	17.91 ± 2.25 (0.16)
t-test value	11.5		10.4		6.4	
P-value	<0.01		<0.01		<0.01	
BMI						
Mean ± SD (SEM)	23.34 ± 4.43 (0.25)	22.31 ± 4.70 (0.22)	23.59 ± 4.66 (0.33)	22.55 ± 5.17 (0.32)	22.87 ± 3.93 (0.38)	21.99 ± 3.97 (0.29)
t-test value	3.04		2.26		1.82	
P-value	0.002		0.025		0.07	

Among the 302 participants with TME, 176 (23.4%) had partial eruption and 126 (16.7%) had complete eruption. The frequencies of participants at different TME statuses differed significantly at various chronological ages (chi-square value 203.9, p < 0.01). At the age of 15 years, 15 (14.4%) participants had partial eruptions, while 2 (1.9%) had total eruptions. Non-eruption of TM was most commonly observed in females (n = 192, 64.6%) and in urban participants (n = 372, 65.0%). With respect to BMI, the underweight category of participants (n = 82, 73.9%) had a higher frequency of non-eruption (χ² = 14.07, p = 0.03) compared to other BMI categories (Table 3).

**Table 3:** Association of third molar eruption status with sociodemographic variables. The data has been represented as frequency (n) and percentage (%); total sample (N) =453. BMI: body mass index

Variable	Categories	Total	Third molar eruption status			Chi-square value	P-value
			Not erupted (n = 451)	Partial eruption (n = 176)	Complete eruption (n = 126)		
Chronological age in years	14	30	30 (100.0%)	0 (0.0%)	0 (0.0%)	203.9	<0.01
	15	104	87 (83.7%)	15 (14.4%)	2 (1.9%)		
	16	91	68 (74.7%)	19 (20.9%)	4 (4.4%)		
	17	145	97 (66.9%)	36 24.8%)	12 (8.3%)		
	18	63	38 (60.3%)	20 (31.7%)	5 (7.9%		
	19	91	46 (50.5%)	25 (27.5%)	20 (22.0%)		
	20	96	45 (46.9%)	27 (28.1%)	24 (25.0%)		
	21	66	27 (40.9%)	17 (25.8%)	22 (33.3%)		
	22	21	8 (38.1%)	7 (33.3%)	6 (28.6%)		
	23	17	1 (5.9%)	3 (17.6%)	13 (76.5%)		
	24	10	0 (0.0%)	5 (50.0%)	5 (50.0%)		
	25	9	1 (11.1%)	0 (0.0%)	8 (88.9%)		
	26	10	3 (30.0%)	2 (20.0%)	5 (50.0%)		
Sex	Male	456	259 (56.8%)	119 (26.1%)	78 (17.1%)	5.6	0.06
	Female	297	192 (64.6%)	57 (19.2%)	48 (16.2%)		
Residential status	Rural	181	79 (43.6%)	51 (28.2%)	51 (28.2%)	31.5	<0.01
	Urban	572	372 (65.0%)	125 (21.9%)	75 (13.1%)		
BMI	Normal	452	263 (58.2%)	107 (23.7%)	82 (18.1%)	14.07	0.03
	Underweight	111	82 (73.9%)	17 (15.3%)	12 (10.8%)		
	Overweight	141	80 (56.7%)	35 (24.8%)	26 (18.4%)		
	Obese	49	26 (53.1%)	17 (34.7%)	6 (12.2%)		

Among male participants, TME status was found to be significantly associated with both chronological age and residential status (chi-square value = 34.3, p < 0.01). Of the 96 males with a chronological age of 15 years, 15 (15.6%) experienced partial eruption, and 2 (2.1%) had total tooth loss due to eruption malformation. Eruption occurred in almost all male participants by the age of 22, except for one. Additionally, the frequency of non-eruption was observed to be substantially higher among urban males (p < 0.01) (Table 4).

**Table 4:** Association of third molar eruption status with sociodemographic variables among males. The data has been represented as frequency (n) and percentage (%); total sample (N) =456. BMI: body mass index

Variable	Categories	Total	Third molar eruption status			Chi-square value	P-value
			Not erupted (n = 259)	Partial eruption (n = 119)	Complete eruption (n = 78)		
Chronological age in years	14	16	16 (100.0%)	0 (0.0%)	0 (0.0%)	153.9	<0.01
	15	96	79 (82.3%)	15 (15.6%)	2 (2.1%)		
	16	62	44 (71.0%)	15 (24.2%)	3 (4.8%)		
	17	72	43 (59.7%)	23 (31.9%)	6 (8.3%)		
	18	36	20 (55.6%)	13 (36.1%)	3 (8.3%)		
	19	43	20 (46.5%)	13 (30.2%)	10 (23.3%)		
	20	51	19 (37.3%)	15 (29.4%)	17 (33.3%)		
	21	40	13 (32.5%)	12 (30.0%)	15 (37.5%)		
	22	14	4 (28.6%)	6 (42.9%)	4 (28.6%)		
	23	12	0 (0.0%)	3 (25.0%)	9 (75.0%)		
	24	5	0 (0.0%)	3 (60.0%)	2 (40.0%)		
	25	6	0 (0.0%)	0 (0.0%)	6 (100.0%)		
	26	3	1 (33.3%)	1 (33.3%)	1 (33.3%)		
Residential status	Rural	114	41 (36.0%)	36 (31.6%)	37 (32.4%)	34.3	<0.01
	Urban	342	218 (63.7%)	83 (24.3%)	41 (12.0%)		
BMI	Normal	260	144 (55.4%)	66 (25.4%)	50 (19.2%)	8.1	0.2
	Underweight	65	44 (67.7%)	11 (16.9%)	10 (15.4%)		
	Overweight	97	54 (55.7%)	29 (29.9%)	14 (14.4%)		
	Obese	34	17 (50.0%)	13 (38.2%)	4 (11.8%)		

TME status was found to be significantly associated with chronological age (chi-square value = 69.74, p < 0.01) and BMI (chi-square value = 12.7, p = 0.048) among female participants. TME was shown to be delayed in females for up to 15 years. Unlike males, no substantial difference (chi-square value = 2.5, p = 0.29) was observed in TME with respect to the residential status of females. Non-eruption of M3 was noted significantly among underweight females (82.6%, n = 38/46) (Table 5).

**Table 5:** Association of third molar eruption status with sociodemographic variables among females. The data has been represented as frequency (n) and percentage (%); total sample (N) =297. BMI: body mass index

Variable	Categories	Total	Third molar eruption status			Chi-square value	P-value
			Not erupted (n = 192)	Partial eruption (n = 57)	Complete eruption (n = 48)		
Chronological age in years	14	14	14 (100.0%)	0 (0.0%)	0 (0.0%)	69.74	<0.01
	15	8	8 (100.0%)	0 (0.0%)	0 (0.0%)		
	16	29	24 (82.8%)	4 (13.8%)	1 (3.4%)		
	17	73	54 (74.0%)	13 (17.8%)	6 (8.2%)		
	18	27	18 (66.7%)	7 (25.9%)	2 (7.4%)		
	19	48	26 (54.2%)	12 (25.0%)	10 (20.8%)		
	20	45	26 (57.8%)	12 (26.7%)	7 (15.6%)		
	21	26	14 (53.8%)	5 (19.2%)	7 (26.9%)		
	22	7	4 (57.1%)	1 (14.3%)	2 (28.6%)		
	23	5	1 (20.0%)	0 (0.0%)	4 (80.0%)		
	24	5	0 (0.0%)	2 (40.0%)	3 (60.0%)		
	25	3	1 (33.3%)	0 (0.0%)	2 (66.7%)		
	26	7	2 (28.6%)	1 (14.3%)	4 (57.1%)		
Residential status	Rural	67	38 (56.7%)	15 (22.4%)	14 (20.9%)	2.5	0.29
	Urban	230	154 (67.0%)	42 (18.3%)	34 (14.8%)		
BMI	Normal	192	119 (62.0%)	41 (21.4%)	32 (16.7%)	12.7	0.048
	Underweight	46	38 (82.6%)	6 (13.0%)	2 (4.3%)		
	Overweight	44	26 (59.1%)	6 (13.6%)	12 (27.3%)		
	Obese	15	9 (60.0%)	4 (26.7%)	2 (13.3%)		

Jaw differences in TME among participants

Clinical investigations have revealed that 787 of the 3012 M3s under review had noticeably erupted. More TMEs were seen in the mandible (457) compared to the maxilla (330). In males, complete mandibular eruption occurred earlier than maxillary eruption, while in females, the opposite was observed. Additionally, the mean age of partial eruption of M3 was earlier in the right quadrant, specifically among males, compared to females (Table 6).

**Table 6:** Mean ages at third molar eruption in the different quadrants of the jaw between genders. SD: standard deviation; SEM: standard error of mean

Quadrants of the jaw	Overall		Male		Female		t-value	P-value
	N	Mean ± SD (SEM)	N	Mean ± SD (SEM)	N	Mean ± SD (SEM)		
Maxillary right	8	17.50 ± 1.93 (0.68)	5	16.60 ± 1.52 (0.68)	3	19.00 ± 1.73 (1.00)	-1.98	0.12
Maxillary left	13	18.15 ± 2.03 (0.56)	8	18.00 ± 2.07 (0.73)	5	18.40 ± 2.19 (0.98)	-0.32	0.75
Maxillary both	21	19.95 ± 2.65 (0.58)	13	20.00 ± 2.27 (0.63)	8	19.88 ± 3.36 (1.19)	0.09	0.93
Mandibular right	26	18.08 ± 2.65 (0.52)	19	17.95 ± 2.99 (0.69)	7	18.43 ± 1.51 (0.57)	-0.54	0.60
Mandibular left	22	18.55 ± 2.36 (0.50)	16	18.81 ± 2.66 (0.67)	6	17.83 ± 1.70 (0.48)	1.19	0.24
Mandibular both	71	18.63 ± 2.15 (0.26)	46	18.30 ± 2.23 (0.33)	25	19.24 ± 1.90 (0.38)	-1.86	0.07
Both right	15	18.13 ± 2.33 (0.60)	12	18.08 ± 2.57 (0.74)	3	18.33 ± 1.15 (0.67)	-0.25	0.81
All	126	20.56 ± 2.60 (0.23)	78	20.49 ± 2.51 (0.28)	48	20.69 ± 2.76 (0.40)	-0.41	0.68

## Discussion

In recent years, age diagnosis has become a demanding task for several reasons, such as disputes with age certificates and the need to authenticate them for various legal and civil concerns, including cases involving illegal unaccompanied minors [11]. Only the development of third molars is taken into consideration to address tooth age in teenagers or people in their 20s and 30s. The other methods for determining age during this time are dubious [12]. There are physiological variations in the age at which teeth erupt due to inherited, dietary, gender, and regional factors [13]. Moreover, due to the extremely high interethnic diversity of M3, formulas used to determine a region’s age based on stages of growth of M3 cannot be applied to other groups [10]. In this context, the present study clinically examined 753 Assamese individuals, comprising both rural and urban participants, to assess the average eruption age of M3 and factors influencing TME.

The mean age of non-eruption was lower for males than for females (16.80 ± 2.11 years vs. 17.91 ± 2.25 years), which agrees with another study [14]. This study recruited 14-year-old individuals to confirm the timing of TME in the local community. Prior radiological investigations among the Indian population have documented that the gingival emergence of M3 appears as early as 14 years [15]. However, none of the 14-year-old individuals demonstrated TME in the current study. Males (n = 197, 43.2%) had substantially more M3 eruptions (p = 0.03) than females (n = 105, 35.4%). M3 eruption statuses of both genders were substantially linked with their chronological age (p < 0.01). The present findings align with the observations of another study [16].

The mean BMI was found to be the highest among males in the M3-erupted group. Underweight females were substantially less likely to have their M3s erupted (82.6%, n = 38/46). The stages of tooth eruption can be accelerated by nutritional status. India continues to have an elevated rate of stunting and underweight among adolescents [17]. The double burden of malnutrition continues to be a serious public health concern, especially for Indian women of reproductive age [18]. The increased incidence of undernutrition among Indian tribes is exacerbated by a strong gender bias [19]. An earlier study also documented a link between BMI and TME [20]. Nutritional status, particularly protein/calorie malnutrition, often leads to delayed tooth eruption, smaller tooth size, less soluble enamel, and malfunctioning salivary glands [21,22].

The non-eruption of M3 was higher among urban individuals (n = 372, 65.0%), especially among urban males (p < 0.01). The growth of the human adult jaw is significantly influenced by nutrition and masticatory processes. Nearly 86% of the Assamese population still resides in rural areas, making the state primarily a rural one. Numerous tribes with diverse customs, cultures, attire, and unique lifestyles may be found in the state. Rural cultures’ non-refined, fibrous diets help the jaw’s trigonal spacing develop, allowing the M3 to erupt [23]. A greater prevalence of M3 impaction has been previously documented among the urban population compared to rural populations [24]. Almost 95.3% of the scheduled tribe population in Assam resides predominantly in rural areas [25]. A recent study documented that M3 non-eruption is more common among the non-tribal population compared to the tribal population [26]. Another study from north-east India also noted variations in the size of the teeth, up to the second molars, between four ethnic groups and within the same population [27]. Both tribal originality and dietary patterns might be attributed to the difference in TME eruption patterns among urban and rural populations. However, more extensive community-based studies are needed to validate these causalities.

Particularly for males compared to females, the mean age of partial eruption of the M3 was earlier in the right quadrant. Females showed an early TME in the maxillary jaw (both) compared to males. At the same time, males showed an early TME in the mandibular jaw (both) compared to females. Previous studies have found that measures of tooth inclination (angles α and β), gonial angle, and space parameters are crucial for forecasting the eruption of the third molar. Third molars that are strongly angulated (>27.0°) seem to have a low probability of eruption in the future and a high likelihood of forming a relationship with the mandibular canal [28,29]. Males exhibited higher mean values of mesiodistal width, lower eruption space measurements, and a β-angle compared to females radiographically [30], which influences an earlier TME in the mandibular jaw among males.

The findings of the present study suggest that the clinical eruption of M3 is substantially correlated to a person’s chronological age and gender. Hence, it may be instrumental to predict a person’s chronological age based on the dental age estimated by TME status among the study population. The BMI of women and the residential status of men were found to be the gender-specific determinants affecting TME among Assamese residents. Future studies are necessary to confirm whether these factors have a causal relationship with TME. However, in clinical settings, practitioners may take these aspects into account while assessing and treating conditions related to third molar eruption and periodontal health issues in the management of specific patients.

Limitations

Only the clinical dental examination method was used in this investigation, which limits our observations to clinical eruption only. The degree of calcification and the developmental stage of M3 were not evaluated using radiological techniques such as X-rays and orthopantomograms. Additionally, neither the radiological assessment of the impaction nor the study of M3 mineralisation was conducted. The lack of an orthopantomogram or cephalometric radiograph, which provides a clear visual representation of tooth growth, was another limitation of this study. Additionally, the cross-sectional design of the study captures eruption status at a single time point, which prevents analysis of progression or causality. Further, single-site sampling might limit the generalizability of the findings.

## Conclusions

Chronological age was observed to be strongly correlated with their TME status in both genders. Among the Assamese population, a significant gender gap exists in TME status. The TME status was found to be influenced by several sociodemographic factors in the study population. Although the current study found a strong correlation between TME and the participants’ BMI and residential status, further research focusing on specific demographics can help validate these findings among the local population.

## References

[1] Marcante B, Marino L, Cattaneo NE, Delicati A, Tozzo P, Caenazzo L (2025). Advancing forensic human chronological age estimation: biochemical, genetic, and epigenetic approaches from the last 15 years: a systematic review. Int J Mol Sci.

[2] Celik S, Zeren C, Celikel A, Yengil E, Altan A (2014). Applicability of the Demirjian method for dental assessment of southern Turkish children. J Forensic Leg Med.

[3] Arany S, Iino M, Yoshioka N (2004). Radiographic survey of third molar development in relation to chronological age among Japanese juveniles. J Forensic Sci.

[4] Liversidge HM, Peariasamy K, Folayan MO (2017). A radiographic study of the mandibular third molar root development in different ethnic groups. J Forensic Odontostomatol.

[5] Kutesa AM, Ndagire B, Nabaggala GS, Mwesigwa CL, Kalyango J, Rwenyonyi CM (2019). Socioeconomic and nutritional factors associated with age of eruption of third molar tooth among Ugandan adolescents. J Forensic Dent Sci.

[6] Almonaitiene R, Balciuniene I, Tutkuviene J (2010). Factors influencing permanent teeth eruption. Part one--general factors. Stomatologija.

[7] Kjær I (2014). Mechanism of human tooth eruption: review article including a new theory for future studies on the eruption process. Scientifica (Cairo).

[8] Al-Batayneh OB, Shaweesh A (2018). Clinical duration of eruption of deciduous teeth in Jordanian children: a cross-sectional study. Arch Oral Biol.

[9] Shaweesh AI (2016). Timing of clinical eruption of third molars in a Jordanian population. Arch Oral Biol.

[10] Khosronejad A, Navabi M, Sakhdari S, Rakhshan V (2017). Correlation between chronological age and third molar developmental stages in an Iranian population (Demirjian method). Dent Res J (Isfahan).

[11] Macha M, Lamba B, Avula JS, Muthineni S, Margana PG, Chitoori P (2017). Estimation of correlation between chronological age, skeletal age and dental age in children- a cross-sectional study. J Clin Diagn Res.

[12] Bhat VJ, Kamath GP (2007). Age estimation from root development of mandibular third molars in comparison with skeletal age of wrist joint. Am J Forensic Med Pathol.

[13] Anu V, Brindha JR, Carol PT, Diana PC, Elsy JD, Garima S (2020). Does body mass index affect tooth eruption sequence? A study among 6-7 years old schoolchildren in Chennai, India. Int J Clin Pediatr Dent.

[14] Priyadharshini KI, Idiculla JJ, Sivapathasundaram B, Mohanbabu V, Augustine D, Patil S (2015). Age estimation using development of third molars in South Indian population: a radiological study. J Int Soc Prev Community Dent.

[15] Putul M, Konwar R, Dutta M, Basumatary B, Rajbongshi MC, Thakuria KD, Sarma B (2021). Assessment of age at the stages of the eruption of third molar teeth among the people of north-eastern India. Biomed Res Int.

[16] Sisman Y, Uysal T, Yagmur F, Ramoglu SI (2007). Third-molar development in relation to chronologic age in Turkish children and young adults. Angle Orthod.

[17] Parida J, Bagepally BS, Patra PK, Pati S, Kaur H, Acharya SK (2025). Prevalence and associated factors of undernutrition among adolescents in India: a systematic review. BMC Public Health.

[18] Surpam S, Dixit P, Ramesh S, Mishra P (2025). Understanding the double burden of malnutrition among women: health implications and challenges. Demography India (RASTA).

[19] Kshatriya GK, Acharya SK (2016). Gender disparities in the prevalence of undernutrition and the higher risk among the young women of Indian tribes. PLoS One.

[20] Lewis JM, Senn DR (2010). Dental age estimation utilizing third molar development: a review of principles, methods, and population studies used in the United States. Forensic Sci Int.

[21] Reis CL, Barbosa MC, Henklein S (2021). Nutritional status is associated with permanent tooth eruption in a group of Brazilian school children. Glob Pediatr Health.

[22] Sheetal A, Hiremath VK, Patil AG, Sajjansetty S, Kumar SR (2013). Malnutrition and its oral outcome - a review. J Clin Diagn Res.

[23] Aher KA, Kadu SS (2019). Pattern of eruption of the third molar teeth in the age group of 17-25 years of medical college students. VIMS Health Sci J.

[24] Venu Gopal Reddy K (2012). Distribution of third molar impactions among rural and urban dwellers in the age group of 22-30 years in South India: a comparative study. J Maxillofac Oral Surg.

[25] Saikia S, Medhi B, Medhi BK (2012). Spatial distribution of tribal population and inter tribal differences in population growth: a critical review on demography and immigration in Assam. IOSR J Humanities Soc Sci.

[26] Kumar KR, Kolay SK (2022). Comparative study of third molar tooth eruption between tribal and non-tribal groups of Bastar Region, Chhattisgarh. Human Biol Rev.

[27] Majumder P, Mahanta P, Kalita C (2022). Variability and patterning in permanent tooth dimensions among four ethnic groups from the north-eastern states of India. Biomed Res Int.

[28] Dzipunova B, Hodzic MJ, Spasova NT (2023). Eruption assessment and potential for impaction of the third mandibular molars - radiographic examination. IOSR J Dent Med Sci.

[29] Vranckx M, Ockerman A, Coucke W (2019). Radiographic prediction of mandibular third molar eruption and mandibular canal involvement based on angulation. Orthod Craniofac Res.

[30] Kaur R, Kumar AC, Garg R, Sharma S, Rastogi T, Gupta VV (2016). Early prediction of mandibular third molar eruption/impaction using linear and angular measurements on digital panoramic radiography: a radiographic study. Indian J Dent.

